# Population Genetic and Functional Analysis of a *cis*-Regulatory Polymorphism in the *Drosophila* *melanogaster* *Metallothionein A* gene

**DOI:** 10.3390/genes10020147

**Published:** 2019-02-14

**Authors:** Timothy J. S. Ramnarine, Amanda Glaser-Schmitt, Ana Catalán, John Parsch

**Affiliations:** 1 Division of Evolutionary Biology, Faculty of Biology, LMU Munich, Grosshaderner Str. 2, 82152 Planegg-Martinsried, Germany; ramnarine@bio.lmu.de (T.J.S.R.); glaser@bio.lmu.de (A.G.-S.); catalan@bio.lmu.de (A.C.); 2 Evolutionary Biology Centre (EBC), Uppsala University, Norbyvägen 14-18 75236, Uppsala, Sweden

**Keywords:** gene expression, *cis*-regulation, deletion, indel, untranslated region, oxidative stress, population genetics, *Drosophila melanogaster*

## Abstract

Although gene expression can vary extensively within and among populations, the genetic basis of this variation and the evolutionary forces that maintain it are largely unknown. In *Drosophila* *melanogaster*, a 49-bp insertion/deletion (indel) polymorphism in the *Metallothionein A* (*MtnA*) gene is associated with variation in *MtnA* expression and oxidative stress tolerance. To better understand the functional and evolutionary significance of this polymorphism, we investigated it in several worldwide populations. In a German population, the deletion was present at a high and stable frequency over multiple seasons and years, and was associated with increased *MtnA* expression. There was, however, no evidence that the polymorphism was maintained by overdominant, seasonally fluctuating, or sexually antagonistic selection. The deletion was rare in a population from the species’ ancestral range in sub-Saharan Africa and is likely the result of non-African admixture, suggesting that it spread to high frequency following the species’ out-of-Africa expansion. Using data from a North American population, we found that the deletion was associated with *MtnA* expression and tolerance to oxidative stress induced by menadione sodium bisulfite. Our results are consistent with the deletion being selectively favored in temperate populations due to the increased *MtnA* expression and oxidative stress tolerance that it confers.

## 1. Introduction

The expansion of a species into new territories provides the opportunity for adaptation to novel environmental conditions. Although such adaptation is of fundamental interest to evolutionary biologists, the identification of locally adaptive traits, along with their underlying molecular and genetic bases, has proven to be challenging. Given its well-understood genetics and experimental tractability, *Drosophila melanogaster* has become a leading model organism for studying the molecular basis of adaptation. These studies typically involve the comparison of DNA sequence and/or gene expression variation between populations from the ancestral (sub-Saharan African) and derived (cosmopolitan) species ranges [1,2,3,4,5,6,7], or among multiple populations spanning an environmental gradient, such as a latitudinal cline [8,9,10]. Clinal variation has been detected for several types of genetic polymorphism in *D. melanogaster*, including chromosomal inversions [9], allozymes [11,12], and single nucleotide polymorphisms (SNPs) [13,14]. Moreover, some genetic variants show repeated oscillations in frequency across seasons in temperate populations [15]. Collectively, these studies suggest that a considerable amount of genetic and expression variation may be maintained within *D. melanogaster* due to local or seasonal adaptation, although detailed functional studies of the underlying causal variants and how they exert their effects on a relevant organismal phenotype remain rare [16,17,18,19,20,21].

**Figure 1:**
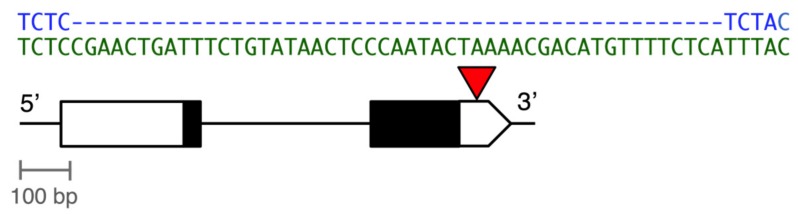
Diagram of the 49-bp insertion/deletion (indel) polymorphism in the 3’ untranslated region (UTR) of the *D. melanogaster Metallothionein A (MtnA)* gene (Chromosome 3R, coordinates 9,783,407–9,784,370). Exons are shown as boxes, with the coding regions in black and the UTRs in white. The red triangle indicates the site of the indel polymorphism, with the DNA sequences of the deletion (blue) and non-deletion (green) variants shown above.

Global populations of *D. melanogaster* are polymorphic for a 49-bp deletion in the 3′ untranslated region (UTR) of the *Metallothionein A* (*MtnA*) gene, as shown in Figure 1 [18,22,23]. This deletion is present at high frequency (34–100%) in cosmopolitan populations but low frequency (0–8%) in sub-Saharan Africa and is absent in other *Drosophila* species [18,23,24], suggesting that it is a derived mutation that has spread to high frequency following the species’ out-of-Africa expansion. Furthermore, the frequency of the deletion increases with distance from the equator in Europe, North America, and Australia, which is consistent with adaptation to temperate environments [18]. The deletion is associated with increased *MtnA* expression and reporter gene experiments have demonstrated that, in a common genetic background, the deletion causes an expression increase in the range of 1.4 to 2.3-fold [18]. The deletion also is associated with increased tolerance to oxidative stress in isofemale lines derived from Europe and Asia [18]. Taken together, these results suggest a scenario in which the *MtnA* 3′ UTR deletion is selectively favored in temperate environments due to the increased oxidative stress tolerance that it confers, which is mediated by higher *MtnA* expression levels. The importance of oxidative stress as a selective factor in temperate populations is further supported by studies of the *Bari-Juvenile hormone epoxy hydrolase (Bari-Jheh)* transposable element insertion, which is present at high frequency in temperate regions and upregulates expression of the *Jheh* genes, leading to increased oxidative stress tolerance [25,26]. In the case of *MtnA*, the 3′ UTR deletion is presumably deleterious under some environmental conditions, which would explain why it remains polymorphic within temperate populations, as well as its rarity in sub-Saharan Africa and other tropical and sub-tropical regions.

In the current study, we determine the frequency of the *MtnA* 3′ UTR insertion/deletion (indel) polymorphism in a large sample of wild-caught flies of both sexes from a temperate European population (Munich, Germany) across seasons and years. This allows us to test for potential forms of balancing selection, such as seasonally fluctuating selection, overdominant selection, or sexual antagonism, which could be involved in the maintenance of this polymorphism. We further examine the effect of the deletion on *MtnA* expression in nearly-isogenic lines derived from this population. We also use publicly available data to test for associations between the deletion, *MtnA* expression, and oxidative stress tolerance in a large North American population sample. Finally, by re-analyzing genome sequence data from a sub-Saharan African population, we determine the frequency of the deletion in the ancestral species range and test whether it is consistent with ancient standing variation or more recent admixture with non-African flies.

## 2. Materials and Methods

### 2.1. Fly Collection and Maintenance

In 2016 and 2017, wild *D. melanogaster* were collected twice per year (late June and early September) in Munich, Germany using traps with banana-yeast bait. After collection, the flies were transferred to individual 35 ml vials containing cornmeal-yeast-molasses medium. To ensure that the wild-caught flies were *D. melanogaster* and not the closely related *D. simulans*, the species identity of all collected males was confirmed by visual inspection of the genitalia under a dissecting microscope. A subset of the wild-caught females (50–60 females per collection) was allowed to lay eggs in the vial and the species identity was confirmed from their male offspring using the above method. We did not detect *D. simulans* in any of the collections. All of the original wild-caught flies from each season were preserved for later genotyping by freezing at –80 °C.

Two isofemale lines (designated here as M9 and M12) collected in Munich in 2014 [27] were found to be polymorphic for the *MtnA* 3’ UTR deletion by genotyping individual offspring (see Section 2.2). These stocks were maintained with random mating for 50–60 generations in order to homogenize the genomic background, then individual males and females were genotyped and mated to generate new inbred lines homozygous for either the insertion or deletion allele. From each of the two original isofemale lines, three replicate insertion and deletion lines were established. These lines were used to test the effect of the indel polymorphism on *MtnA* expression in a nearly-isogenic background. All stocks were maintained on cornmeal-yeast-molasses medium at 21 °C and a 14 hour light/10 hour dark cycle.

### 2.2. Detection of the MtnA Deletion in Wild-Caught Flies

The *MtnA* genotype of individual flies was determined following the method of Catalán et al. [18]. Briefly, genomic DNA was extracted from individual flies according to the MasterPure Complete DNA and RNA purification kit (Epicenter, Madison, WI, USA) protocol. PCR was performed using the primers 5’-GCCGCAGACCAATTGATTA-3’ and 5’-TTCTTTCCAGGATGCAAATG-3’, which amplify fragments of 254 bp (deletion) or 303 bp (non-deletion), or the primers 5’-GCCGCAGACCAATTGATTA-3’ and 5’-TTCTTTCCAGGATGCAAATG-3’, which amplify fragments of 485 bp (deletion) or 534 bp (non-deletion). The thermal cycling conditions were as follows: denaturation at 98 °C for 30 sec, 35 cycles of 98 °C for 5 sec, and 60 °C for 10 sec, and extension at 72 °C for 2 min. Following PCR, the genotype was determined from the banding pattern on a 1.5% agarose electrophoresis gel.

### 2.3. Quantitative Reverse Transcription PCR

For each biological replicate, total RNA was extracted from the heads of 10 males (aged 4–6 days) following the MasterPure Complete DNA and RNA purification kit (Epicenter) protocol, including DNase I digestion. We focused on male heads, as this sex and tissue combination showed a consistent effect of the *MtnA* 3’ UTR deletion on reporter gene expression in a previous study [18]. For each sample, 650 ng of total RNA was used for cDNA synthesis with SuperScript III reverse transcriptase (Invitrogen, Carlsbad, CA, USA) and random hexamer primers following the manufacturer’s protocol. Residual RNA was removed by incubating with RNase H (New England BioLabs, Ipswich, MA, USA). Quantitative PCR was carried out with 25 ng of cDNA per reaction and SsoAdvanced Universal SYBR Green Supermix (Bio-Rad, Hercules, CA, USA) according to the manufacturer’s guidelines. The following *MtnA* primers, which amplify a cDNA fragment of 93 bp were used: 5’-GCACTTGCAGTCAGATCC -3’ and 5’- TCAATCAAGATGCCTTGCC-3’. For each sample, the ribosomal protein gene *RpL32* was also amplified as a reference using the primers 5’-AGCATACAGGCCCAAGATCG-3’ and 5’-AGCATACAGGCCCAAGATCG-3’, which amplify a fragment of 113 bp [28]. The following cycling conditions were used: 95 °C for 30 sec, 35 cycles of 95 °C for 10 sec, and 63 °C for 30 sec. Amplification products were quantified using a CFX96 real-time thermal cycler (Bio-Rad). For each nearly-isogenic background (M9 or M12) and genotype (deletion or non-deletion), 25–28 biological replicates were performed, each with two technical replicates (i.e., starting from the same cDNA). To compare *MtnA* expression among samples, the ΔΔCt method was used, with *RpL32* as the reference [29]. The effect of the deletion on expression was tested using a two-factor ANOVA (background and genotype) as implemented in R [30].

### 2.4. Detection of the MtnA Deletion using Genome Sequence Data

Raw genomic sequence reads (150 bp, paired-end) of 193 *D. melanogaster* lines collected in Siavonga, Zambia [31,32,33] and 17 lines collected in Kafue National Park, Zambia [34] were downloaded from the NCBI sequence read archive (SRA; accession numbers SRP006733 and PRJNA329555, respectively). For each line, 150 bp, paired-end reads were generated from haploid embryos [35]. Thus, we expect to detect only one allele per line. The reads were mapped to a reference file containing sequences of both the deletion and non-deletion alleles of the *MtnA* 3’ UTR using NextGenMap [36]. The deletion reference sequence consisted of 90 bp (49 bp before and 41 bp after the deletion site), while the non-deletion reference sequence contained the same 90 bp, plus an additional 49 bp at the deletion site. To determine the *MtnA* genotype, we required that reads mapping to one of the two reference sequences overlap for at least 15 bp on either side of the deletion site. With this approach, we could unambiguously classify the *MtnA* allele of 209 out of 210 lines. For one of the Siavonga lines, there was a single read matching the deletion reference and 18 reads matching the non-deletion reference. This line was excluded from further analysis. 

Raw genomic reads of 185 lines from Raleigh, North Carolina, USA were downloaded from the SRA (accession PRJNA36679). These lines represent the *Drosophila* Genetic Reference Panel (DGRP) and are maintained as highly inbred lines that have been used for multiple studies of quantitative morphological, physiological, behavioral, and gene expression traits [37,38]. The sequences were a mixture of single-end and paired-end reads and ranged in length from 45 to 126 bp. To determine the *MtnA* genotype of each line, we used the mapping procedure described above, but due to the shorter read lengths, required a minimum overlap of only 8 bp on either side of the deletion site. With this approach, we could classify the *MtnA* allele of 163 lines (at least 95% of all reads mapping to one allele). The remaining 22 lines had reads mapping to both the deletion and non-deletion references, with the minor allele at a frequency greater than 5%. Because sequencing was performed using DNA extracted from a pooled sample of 500–1000 flies per line [37,38], it is possible that this ambiguity was caused by residual polymorphism segregating within some of the DGRP lines. These lines were excluded from further analysis.

### 2.5 Detection of Non-African Admixture

To investigate whether the presence of the *MtnA* 3’ UTR deletion in the Zambian population was better explained by ancestral polymorphism or more recent non-African admixture, we constructed a neighbor-joining tree for a 15 kb region surrounding *MtnA* using 20 Zambian sequences (five of which contained the deletion) and 20 sequences from Lyon, France [32,33]. Only aligned nucleotides were considered for tree construction (i.e., indels were excluded) using MEGA 7.0.26 [39]. Node support was assessed by doing 1000 bootstrap iterations [40]. The tree was rooted using the reference sequences of *D. sechellia* and *D. simulans* as outgroups [41] and edited using the R package APE 5.2 [42].

### 2.6. Analysis of MtnA expression and oxidative stress tolerance in DGRP lines

Whole-genome expression data for 185 DGRP lines and oxidative stress tolerance data for 167 DGRP lines were downloaded from the DGRP2 website [43]. Gene expression was measured in two replicates per sex using Affymetrix *Drosophila* 2.0R Tiling Arrays [44]. Oxidative stress tolerance was measured as survival in hours on two separate oxidative stress-inducing agents: paraquat and menadione sodium bisulfite (MSB), a water-soluble derivative of menadione [45]. DGRP lines with only one reported *MtnA* expression replicate in either sex were excluded from all analyses and only lines with both *MtnA* expression and oxidative stress data were included in the oxidative stress analysis. This resulted in a total of 161 lines for the *MtnA* expression analysis and 146 lines for the oxidative stress tolerance analysis. Differences in *MtnA* expression between deletion and non-deletion lines were tested using a Student’s *t* test. The association between oxidative stress tolerance and *MtnA* expression was assessed using Spearman’s rank correlation. The association between *MtnA* 3’ UTR genotype and survival on each oxidative stress-inducing agent was tested using a Cox proportional hazards model [46] with sex, genotype, and line as factors as implemented in the “survival” package [47] in *R* [30].

## 3. Results

### 3.1. The Deletion is Present at a Stable, High Frequency in a European Population

Based on a sample of 11 isofemale lines collected in 2005, Catalán et al. [18] estimated the *MtnA* 3’ UTR deletion frequency in a population from Munich, Germany to be 0.91 with a 95% confidence interval (CI) of 0.73–0.98. In order to refine this estimate and determine if the deletion frequency varies across years or seasons, or between males and females, we collected and genotyped wild *D. melanogaster* from Munich twice per year (late June and early September) in 2016 and 2017. In total, we genotyped 510 wild-caught flies (1020 chromosomes) and found the *MtnA* deletion frequency to be 0.91 with a 95% CI of 0.89­–0.92, which did not differ significantly from the 2005 estimate (Fisher exact test, *P* = 0.99). Thus, the deletion frequency has remained remarkably stable for more than a decade. Similarly, the deletion frequency did not differ between 2016 and 2017, or between the June and September collection of each year (Fisher exact test, *P* > 0.65 in each year) as shown in Table 1. There was also no difference in the frequency of the deletion between males and females (*P* > 0.36 in each season) as shown in Table 1. Thus, there was no evidence for seasonally fluctuating selection or sexually antagonistic selection acting on the indel polymorphism.

Because we genotyped wild-caught flies, we were also able to compare genotype and allele frequencies and test for departures from Hardy–Weinberg equilibrium (HWE), which might be indicative of overdominant selection (heterozygote advantage). In all cases, the genotype frequencies were consistent with the allele frequencies and there were no significant differences from the expectations of HWE, as shown in Table 2, suggesting that overdominant selection does not play a role in maintaining the indel polymorphism. 

### 3.2. The Deletion Leads to Higher MtnA Expression in a Common, European Background

**Figure 2:**
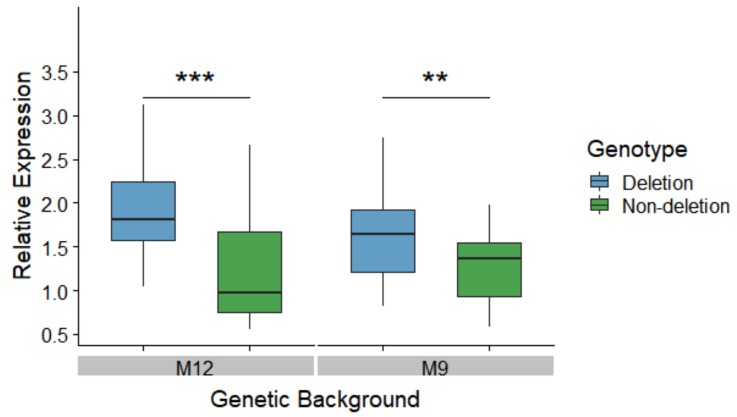
Effect of the indel polymorphism on *MtnA* expression in nearly-isogenic lines within two genetic backgrounds (M12 and M9) derived from Munich, Germany. Expression was measured in male heads by qRT-PCR using the ribosomal protein gene *RpL32* as a reference. Differences in expression were tested with a two-factor ANOVA. **P < 0.01, ***P < 0.005.

To test the effect of the 3’ UTR deletion on *MtnA* expression in a common genetic background, we generated nearly-isogenic lines homozygous for the deletion or non-deletion alleles within the genetic backgrounds of two isofemale lines from Munich, Germany (designated as M9 and M12). Expression was measured in male heads by quantitative reverse transcription PCR (qRT-PCR). Within each of these backgrounds, the deletion lines had significantly higher *MtnA* expression than the non-deletion lines, as shown in Figure 2. The difference in expression was greater in the M12 background (1.85-fold) than in the M9 background (1.20-fold), which might be attributable to variation at other loci segregating between these two backgrounds. However, we did not detect a significant interaction between background and genotype in our analysis (two-factor ANOVA, *P* = 0.29). 

### 3.3. Association of the Deletion with MtnA Expression and Oxidative Stress Tolerance in a North American Population

**Figure 3:**
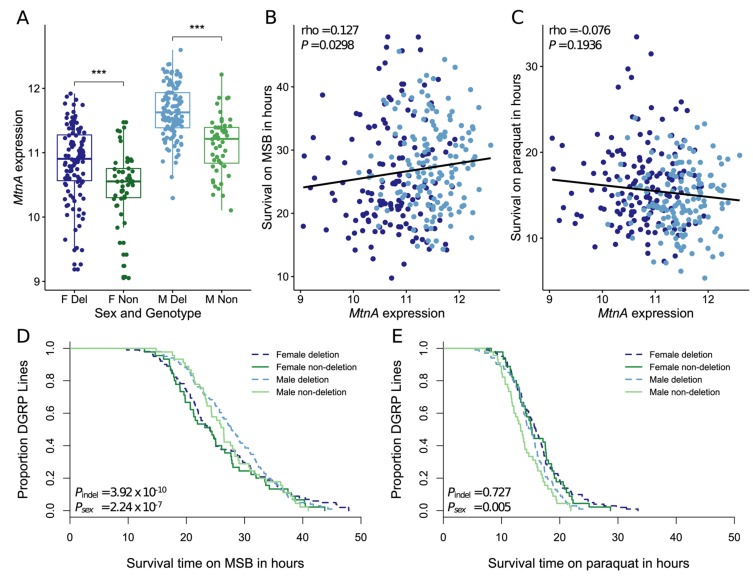
*MtnA* expression and oxidative stress tolerance in *Drosophila* Genetic Reference Panel (DGRP) lines. (**A**) *MtnA* expression in deletion (Del) and non-deletion (Non) lines in females (F) and males (M). Significance was assessed using a t-test. ***P < 0.005. Expression is reported as the log_2_ relative signal intensity from Affymetrix microarrays. (**B,C**). Correlation between *MtnA* expression and survival on menadione sodium bisulfite (MSB) (panel B) and paraquat (panel C). Significance was assessed with Spearman’s rank correlation. Female data points are shown in dark blue and male data points in light blue. A linear regression line is shown in black. (**D,E**). Survival of DGRP lines on MSB (panel D) and paraquat (panel E). Significance was assessed using a Cox proportional hazards model with sex, *MtnA* indel variant, and isofemale line as factors. (**A,D,E**). Female deletion lines are shown in dark blue, male deletion lines in light blue, female non-deletion lines in dark green, and male non-deletion lines in light green.

In order to determine the effect of the indel polymorphism on *MtnA* expression in a North American population, we used genomic and transcriptomic data from the DGRP [37,38]. By re-mapping genomic sequence reads, we were able to determine the *MtnA* indel genotype of 161 DGRP lines for which expression data from whole males and females were available [44]. Of these 161 lines, 112 contained the *MtnA* 3’ UTR deletion. In both sexes, *MtnA* expression was significantly higher in the deletion lines than in the non-deletion lines, as shown in Figure 3A. The deletion was associated with a 1.33-fold increase in *MtnA* expression in females, and a 1.40-fold increase in males. In females, the indel polymorphism accounted for 7.96% of the overall *MtnA* expression variance, while in males it accounted for 22.75%. On average, *MtnA* expression was 1.67-fold higher in males than in females, which is consistent with previous reports of this gene having male-biased expression [48].

It was previously shown that the *MtnA* 3’ UTR deletion was associated with increased oxidative stress tolerance in isofemale lines from Europe and Asia [18]. To test for an association in the DGRP lines, we used data from Weber et al. [45], who measured survival on two oxidative stress-inducing agents (paraquat and MSB). *MtnA* expression was significantly correlated with survival on MSB, as shown in Figure 3B, but not paraquat, as shown in Figure 3C. Additionally, the presence of the deletion was significantly associated with longer survival on MSB, as shown in Figure 3D, but not paraquat, as shown in Figure 3E. Thus, increased *MtnA* expression and the presence of the 3’ UTR deletion, which is associated with increased *MtnA* expression, are both associated with increased tolerance to oxidative stress induced by MSB but not paraquat. Consistent with the male-biased expression of *MtnA*, sex had a highly significant effect on survival on MSB, with males surviving longer than females, as shown in Figure 3D.

### 3.4. The Deletion is Rare in Sub-Saharan Africa and is a Result of European Admixture

**Figure 4:**
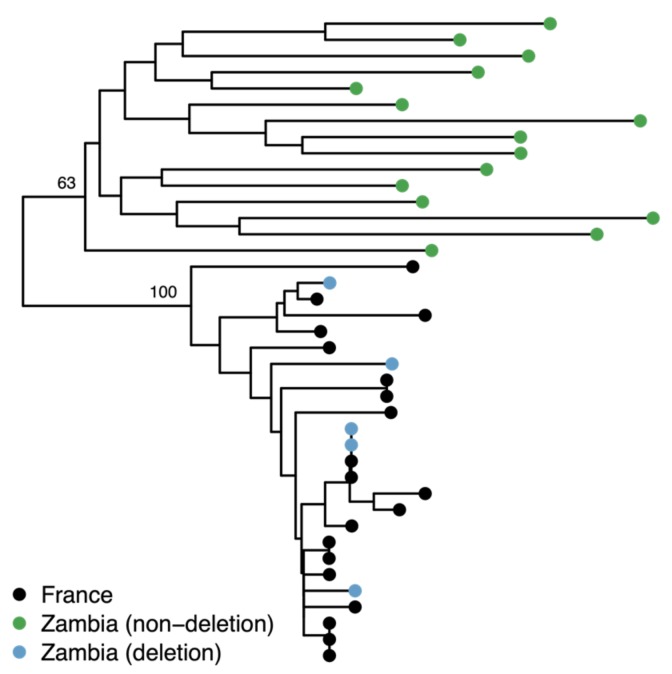
Neighbor-joining tree of the 15 kb region surrounding the *MtnA* gene for 20 Zambian and 20 French sequences. The Zambian sequences containing the *MtnA* 3’ UTR deletion (blue) cluster together with the French sequences (black) with 100% bootstrap support, which is consistent with recent admixture in this region of the genome. The tree was rooted using *D. sechellia* and *D. simulans* as outgroups.

Catalán et al. [18] genotyped 10 isofemale lines collected in Siavonga, Zambia and found the *MtnA* 3’ UTR deletion to be present in a heterozygous state in one line, leading to a frequency estimate of 0.05 (95% CI = 0.01–0.24) and suggesting that the deletion may reflect standing variation present in the ancestral population of *D. melanogaster*. To further investigate this possibility, we determined the *MtnA* genotype of 192 isofemale lines from the Siavonga population using publicly available genome sequence data generated from haploid embryos [32,33]. The deletion was found to be present in five of the genomes, leading to a frequency estimate of 0.026 (95% CI = 0.009–0.060). However, three of the five deletion lines were previously flagged by Lack et al. [32] as showing evidence of non-African admixture within a region of chromosome arm 3R that includes the *MtnA* locus. Thus, it is possible that the presence of the deletion in sub-Saharan Africa is the result of recent admixture and not ancestral variation. To test this, we used SNP data from the *MtnA* region to determine the relationship among the Zambian lines with and without the deletion, as well as with a sample of lines from Europe (Lyon, France). Our results indicated that the Zambian lines with the deletion clustered together with the French lines, while all the Siavonga lines lacking the deletion clustered together in a separate, divergent clade, as shown in Figure 4. Thus, the presence of the deletion in the Zambian population appears to be the result of recent non-African admixture. Consistent with this interpretation, the deletion was not found in a population from a wild African environment (Kafue National Park, Zambia), although only 17 genomes are currently available from this population [34].

## 4. Discussion

By genotyping wild-caught *D. melanogaster* from a European population and re-analyzing publicly available genomic data from an African and a North American population, we were able to gain a better functional and population genetic understanding of the *MtnA* 3’ UTR indel polymorphism. Consistent with a previous study [18], we find that the deletion allele is present at high frequency in cosmopolitan populations, but low frequency within sub-Saharan Africa. In the few sub-Saharan African lines that had the deletion, it was embedded within a genomic region sharing high sequence similarity with European lines, as shown in Figure 4. Thus, the deletion is unlikely to represent standing genetic variation in the ancestral population, but instead a new mutation that increased in frequency following the species’ out-of-Africa migration. In a German population, the deletion appears to have remained at a remarkably stable frequency of approximately 90% for over a decade. However, despite its stable frequency and clinal distribution across continents, we found no evidence for balancing selection acting on the indel polymorphism in this population. The deletion did not vary significantly in frequency across years or seasons, or between the sexes, as shown in Table 1. Thus, there was no evidence for seasonally fluctuating or sexually antagonistic selection acting on this polymorphism. Similarly, we did not detect an excess of heterozygotes, as would be expected for overdominant selection, as shown in Table 2.

The above findings suggest that the *MtnA* indel polymorphism has been subjected to a more complex form of selection. One possibility is that the polymorphism affects a fitness component other than viability, which could not be detected by measuring allele frequencies in adult flies, even with large sample sizes. Another possibility is that the deletion is consistently favored by positive selection in the German population but remains polymorphic in this population due to repeated migration of flies from more tropical populations where the deletion is at intermediate frequency (45–65%) [18] and may be under balancing selection. A third possibility is that *MtnA* is just one of many loci involved in polygenic adaptation. In a scenario where multiple genetic variants of small effect influence a selected trait, alleles may rise in frequency rapidly, but then level off at an intermediate frequency without going to fixation in the population [49]. Our functional analyses are consistent with this interpretation: the two traits we investigated, gene expression and oxidative stress tolerance, both showed evidence of being influenced by variation at multiple loci. In terms of *MtnA* gene expression, the use of nearly-isogenic lines allowed us to determine the effect of the indel polymorphism while minimizing the contribution of other variants across the genome. This indicated that the magnitude of the effect of the indel polymorphism differed depending on the genomic background, as shown in Figure 2. Furthermore, among the DGRP lines, the indel explained only 8–23% of the observed *MtnA* expression variation. Similarly, oxidative stress tolerance is influenced by many loci: 154 SNP loci showed a significant association with survival on MSB in a previous study of the DGRP lines [45]. The *MtnA* gene was not among the candidates influencing MSB tolerance in the previous study. However, that study focused only on SNP markers and, thus, did not consider the *MtnA* indel polymorphism, which does not show strong linkage to SNPs in this region of the genome [18]. The previous study also found susceptibility to oxidative stress to be sexually dimorphic, with many variants displaying sexually antagonistic effects [45]. While we found evidence that the magnitude of the effect of the indel polymorphism on both *MtnA* expression and oxidative stress tolerance was sex-dependent, as shown in Figure 3, the response was in the same direction in males and females, suggesting that the polymorphism has a concordant effect on fitness in both sexes. This is in agreement with there being no evidence for sexual antagonism influencing the frequency of the polymorphism in adult flies, as shown in Table 1.

Catalán et al. [18] found that increased *MtnA* expression, as well as the presence of the *MtnA* 3’ UTR deletion, was associated with increased survival in the presence of hydrogen peroxide, a reactive oxygen species (ROS). While ROS are natural products of metabolism and serve important functions in the cell, high levels, which can be introduced by environmental factors such as UV light or toxins, lead to oxidative stress. Organisms cope with oxidative stress through the induction of antioxidant or free radical scavenger genes, as well as other mechanisms such as apoptosis and cell cycle arrest [50,51]. Thus, it is unsurprising that previous studies found that the transcriptional response to oxidative stress depends on the stress-inducing agent [50,52,53] and that genes associated with oxidative stress tolerance are typically specific to the agent of induction [45]. This is thought to be a product of the different mechanisms of action these agents employ that result in toxicity [45]. Our results provide insight into the mechanism through which *MtnA* expression improves oxidative stress tolerance. We found that increased *MtnA* expression, as well as the *MtnA* deletion allele, is associated with increased tolerance to MSB, but not paraquat, as shown in Figure 3. While paraquat toxicity is primarily driven by redox cycling, MSB toxicity primarily occurs through other reactions with biomolecules unrelated to superoxide formation [54]. It has been suggested that metallothioneins may play a role as scavengers of free radicals in the oxidative stress response [55], which is in line with our findings.

## Figures and Tables

**Table 1:** Frequency of the *MtnA* 3’ UTR deletion across seasons and sexes.

Collection	*N*_female_	Freq_female_ (95% CI)	*N*_male_	Freq_male_ (95% CI)
June 2016	224	0.906 (0.860–0.941)	164	0.902 (0.846–0.943)
Sept 2016	192	0.927 (0.881–0.960)	94	0.894 (0.813–0.948)
June 2017	144	0.903 (0.842–0.946)	174	0.902 (0.848–0.942)
Sept 2017	176	0.909 (0.857–0.947)	60	0.933 (0.838–0.982)

*N* = number of chromosomes; CI = confidence interval. *MtnA: Metallothionein A*; UTR: untranslated region.

**Table 2:** Genotype counts of the *MtnA* 3’ UTR indel polymorphism.

Collection	Del/Del	Del/Non	Non/Non	*P*_HWE_
June 2016	160	31	3	0.731
Sept 2016	120	22	1	0.999
June 2017	131	25	3	0.653
Sept 2017	99	18	1	0.999
All	510	96	8	0.338

Del = deletion allele; Non = non-deletion allele; *P*_HWE_ = *P*-value of a chi-squared test (observed vs. expected) of Hardy–Weinberg equilibrium.
